# A risk-scoring system to predict dupilumab-associated ocular surface disease in patients with atopic dermatitis

**DOI:** 10.3389/fphar.2024.1425550

**Published:** 2024-08-01

**Authors:** Sunyoung Shim, Jung Sun Kim, Jeong Yee, Hye Sun Gwak

**Affiliations:** ^1^ College of Pharmacy and Graduate School of Pharmaceutical Sciences, Ewha Womans University, Seoul, Republic of Korea; ^2^ Department of Pharmacy, Yeouido St. Mary’s Hospital, The Catholic University of Korea, Seoul, Republic of Korea; ^3^ School of Pharmacy, Sungkyunkwan University, Suwon, Republic of Korea

**Keywords:** dupilumab, dupilumab-associated ocular surface disease, DAOSD, atopic dermatitis, conjunctivitis

## Abstract

**Introduction:**

Dupilumab is the first biological treatment for atopic dermatitis (AD). Dupilumab-associated ocular surface disease (DAOSD) is one of the most commonly reported side effects in patients with AD during dupilumab treatment. This study aimed to identify risk factors for DAOSD in a real-world setting and construct a risk-scoring system for predicting DAOSD risk.

**Methods:**

A retrospective analysis was conducted for dupilumab-treated adult patients with AD between April 2019 and September 2023 at Yeouido St. Mary’s Hospital in Korea. Patients aged ≥18 years who received dupilumab to treat AD were included. Univariate and multivariable logistic regression analyses were performed to determine independent risk factors for DAOSD. A risk scoring system was constructed to predict DAOSD risk based on the adjusted odd ratios of significant variables.

**Results:**

Of the 97 dupilumab-treated patients, 28 (28.9%) developed DAOSD. Among them, three (10.7%) patients discontinued dupilumab due to ocular side effects. In the multivariable analysis, older age, history of conjunctivitis, and a baseline Eczema Area and Severity Index (EASI) score ≥28 were independent risk factors for developing DAOSD. Using these variables, a risk-scoring system was constructed. The predicted DAOSD risks for AD patients with 0, 1, 2, 3, 4, and 5 points were 5.8%, 14.2%, 30.7%, 54.3%, 76.2%, and 89.6%, respectively.

**Conclusion:**

In this study, the patient’s age, history of conjunctivitis, and higher baseline EASI score were significantly associated with DAOSD. This risk-scoring system would help identify high-risk patients requiring more caution when initiating dupilumab treatment.

## Introduction

Atopic dermatitis (AD) is a chronic inflammatory skin disorder involving multiple immune pathways (Langan et al., 2020). The incidence and prevalence of AD have increased over previous decades, affecting about 20% of children and up to 10% of adults in high-income countries (Laughter et al., 2021; Stander, 2021). Type 2 helper T cell (Th2) immune responses are considered the key pathway leading to cutaneous inflammation in AD (Bieber, 2022). Th2 biomarkers such as interleukin (IL)-4 and IL-13 are associated with activation of the Th2-dominant immune response, B-cell maturation, immunoglobulin E (IgE) production and reduction in filaggrin gene expression with subsequent disruption of the skin’s barrier function (Seegraber et al., 2018; Aszodi et al., 2019). Managing AD is complex and typically involves a combination of therapeutic strategies, including topical corticosteroids, immunomodulatory agents, and systemic medications (Siegels et al., 2021).

Dupilumab was the first FDA-approved biological treatment for patients with moderate-to-severe AD in 2017 (Strowd and Feldman, 2017). As a fully human monoclonal antibody, it binds to IL-4Rα and inhibits IL-4 and IL-13 signaling, thereby reducing the Th2 response (Gooderham et al., 2018). In many clinical trials and meta-analyses, the efficacy of monotherapy or combination therapy with topical corticosteroids has been proven in adult patients (Simpson et al., 2016; Blauvelt et al., 2017; Halling et al., 2021). Additionally, recent studies have confirmed its clinical effectiveness in children and adolescents (Napolitano et al., 2022a; Napolitano et al., 2022b).

Dupilumab has previously demonstrated an acceptable safety profile (Simpson et al., 2016; Blauvelt et al., 2017). However, common adverse events include injection-site reactions, head and neck dermatitis, hypereosinophilia, arthritis, and ophthalmic complications (Kychygina et al., 2022). Specifically, dupilumab-associated ocular surface disease (DAOSD), including conjunctivitis, blepharitis, and dry eyes, was reported in 10%–50% of AD patients (Bohner et al., 2021; Chen et al., 2021; Halling et al., 2021; Achten et al., 2024). Even though most studies reported that conjunctivitis was treatable, DAOSD was the main reason for discontinuing dupilumab treatment (Halling et al., 2021).

The underlying mechanism of DAOSD remains unclear. However, one proposed mechanism is that dupilumab may cause goblet cell hypoplasia and decrease mucin production by blocking IL-13, resulting in conjunctival inflammation (Bakker et al., 2019). Previous research has identified various risk factors associated with the development of DAOSD, including the AD severity at the initiation of dupilumab treatment, the presence of additional allergic conditions, and a history of keratoconjunctivitis (Thyssen et al., 2017; Treister et al., 2018; Nahum et al., 2020). Nevertheless, conclusive data still needs to be obtained, and there is no risk-scoring system available to predict the DAOSD. Since early recognition and timely referral to ophthalmologists for ocular disease in dupilumab-treated AD patients can reduce the severity of DAOSD (Achten et al., 2023), this study aimed to identify risk factors for developing DAOSD in a real-world setting and proposes a risk-scoring system for DAOSD in patients with AD.

## Materials and methods

### Study participants

AD patients who received dupilumab between April 2019 and September 2023 at Yeouido St. Mary’s Hospital (The Catholic University of Korea, Seoul, Republic of Korea) were retrospectively analyzed. Adult patients (≥18 years) who received dupilumab with an indication of AD were included. In this analysis, patients who met any of the following criteria were excluded: those who 1) received a single dose, 2) received dupilumab at irregular intervals, 3) had chronic conjunctivitis at enrollment, or 4) self-reported ocular symptoms without diagnosis. This study protocol received approval from the Institutional Review Board (IRB) of Yeouido St. Mary’s Hospital (IRB number: SC23RASI0177), and informed consent was waived due to its retrospective nature. Additionally, adherence to STROBE guidelines ensured the comprehensive and rigorous reporting of the findings.

### Dupilumab administration and assessment

A loading dose of 600 mg dupilumab was administered initially, followed by a subcutaneous injection of 300 mg every other week. The AD severity was evaluated by a dermatologist using the Eczema Area and Severity Index (EASI) score, which categorized AD severity into mild (0–5.9), moderate (6.0–22.9), and severe AD (23.0–72.0). DAOSD was defined as the presence of blepharitis, conjunctivitis, keratoconjunctivitis, keratitis, or dry eye syndrome, diagnosed or documented in the electronic medical record (EMR) by a dermatologist or ophthalmologist after starting dupilumab treatment.

### Data collection

Demographic and clinical data of patients were collected from EMR, including gender, age, alcohol consumption, smoking status, comorbidities, and comedications (topical corticosteroids, topical calcineurin inhibitors, oral corticosteroids). Additionally, data on previous AD treatment, and history of allergic comorbidities (allergic rhinitis, asthma, food allergy, conjunctivitis) were obtained. Key clinical indicators were gathered, including EASI scores, total serum IgE levels, and blood eosinophils at baseline. Furthermore, comprehensive details of dupilumab administration data, including dose, interval, and duration, were recorded, along with the reason for discontinuation, DAOSD onset date, symptoms, and management strategies for DAOSD.

### Statistical analysis

Categorical variables were analyzed using Fisher’s exact or chi-squared tests, while quantitative variables were analyzed using the Mann–Whitney or Student’s t-tests. Quantitative variables were converted to binary variables based on predefined cut-off points that satisfy maximum sensitivity and specificity with strong clinical relevance. The cut-off point selection was conducted through receiver operating characteristic (ROC) curve analysis using the “pROC” package in R (v1.18.5) (Robin et al., 2011). For sensitivity analysis, missing values were imputed by using the “mice” package in R (v3.16.0) (van Buuren and Groothuis-Oudshoorn, 2011).

Variables with *p* < 0.05 in the univariate analysis, along with possible confounders (age and gender), were included in the multivariable logistic regression analysis. Backward elimination was used, where variables were included when the *p*-value was less than 0.05 and excluded when the *p*-value was greater than 0.1. From univariate and multivariable analyses, the unadjusted odds ratio (OR) and adjusted OR (aOR) with the 95% confidence intervals (CIs) were calculated, respectively. Using the Hosmer–Lemeshow goodness-of-fit test and the area under the receiver operating characteristic curve (AUROC), the fit and discrimination of the prediction model were determined, respectively.

A risk scoring system was developed based on the multivariable logistic regression model. Each coefficient from the multivariable logistic regression model was divided by the smallest one and rounded to the nearest integer to calculate the risk score. The predicted risk was estimated using logistic regression analysis and compared with the observed risk.

All statistical tests were performed using the SPSS version 20.0 (IBM Corp., Armonk, NY, United States) and R software version 4.3.2 (R Foundation for Statistical Computing, Vienna, Austria). *P*-values <0.05 were considered statistically significant.

## Results

Among the 108 patients enrolled, 11 were excluded for the following reasons: Two were administered a single dose of dupilumab, five were administered at irregular intervals, three had chronic conjunctivitis and one self-reported ocular symptoms. Consequently, a total of 97 AD patients who were treated with dupilumab were eligible for this study. Among them, 28 patients (28.9%) developed DAOSD. Ocular complaints included itching (32.1%), hyperemia (10.7%), eye dryness (7.1%), foreign body sensation (7.1%), pain (3.6%), and eye discomfort (3.6%). The mean duration to the onset of DAOSD was 19.7 ± 23.5 weeks, and DAOSD manifestations were as follows: Conjunctivitis (78.6%), dry eye syndrome (35.7%), keratitis (28.6%), blepharitis (25.0%), and keratoconjunctivitis (17.9%). Among them, three (10.7%) patients discontinued dupilumab due to ocular side effects. Patients who developed DAOSD were prescribed olopatadine 0.7% eye drops (35.7%), fluorometholone 0.1% eye drops (28.6%), and sodium hyaluronate (21.4%) or sodium carboxymethyl cellulose (14.3%). Nine patients were prescribed Maxitrol^®^ eye ointment (dexamethasone, neomycin, and polymyxin B sulfate).

Table 1 presents the demographic and clinical characteristics of enrolled patients at baseline. The median age of dupilumab initiation was 32.4 years, with an interquartile range (IQR) of 24.3–39.9 years, and approximately one-third were women. All patients were classified into severe AD, with an EASI score ranging from 23 to 64. For the history of allergic comorbidities, 69.1% of patients had allergic rhinitis, which was the largest, followed by food allergy (48.5%) and conjunctivitis (18.6%).

**TABLE 1:** Demographic and clinical characteristics of patients included.

Variables	DAOSD (n = 28)	No DAOSD (n = 69)	*p*-value
Gender			0.716
Women	10 (35.7)	22 (31.9)	
Men	18 (64.3)	47 (68.1)	
Age (years)			0.046
<25	4 (14.3)	27 (39.1)	
25–39	14 (50.0)	28 (40.6)	
≥40	10 (35.7)	14 (20.3)	
Smoking	3 (10.7)	7 (10.1)	0.933
Alcohol	7 (25.0)	12 (17.4)	0.392
Comorbidities			
Hypertension	3 (10.7)	10 (14.5)	0.621
Diabetes mellitus	2 (7.1)	5 (7.2)	0.986
Dyslipidemia	8 (28.6)	12 (17.4)	0.217
Cancer	2 (7.1)	3 (4.3)	0.573
History of allergic diseases			
Asthma	6 (21.4)	9 (13.0)	0.301
Allergic rhinitis	21 (75.0)	46 (66.7)	0.421
Food allergy	14 (50.0)	33 (47.8)	0.846
Conjunctivitis	12 (42.9)	6 (8.7)	<0.001
EASI score (mean ± SD)^a^	30.3 ± 8.0	26.9 ± 3.2	0.044
<28	11 (40.7)	48 (70.6)	0.007
≥28	16 (59.3)	20 (29.4)	
IgE (IU/ml, mean ± SD)^b^	8,358.1 ± 9,447.1	3,585.5 ± 4,220.4	0.059
<2,600	4 (23.5)	24 (53.3)	0.035
≥2,600	13 (76.5)	21 (46.7)	
Eosinophils (%, mean ± SD)^c^	9.9 ± 9.6	5.8 ± 4.5	0.053
<5.0	8 (33.3)	29 (50.0)	0.168
≥5.0	16 (66.7)	29 (50.0)	
Previous medications			0.211
Cyclosporine	19 (67.9)	54 (78.3)	
Methotrexate	7 (25.0)	14 (20.3)	
Omalizumab	1 (3.6)	0 (0.0)	
None	1 (3.6)	0 (0.0)	
Unknown	0 (0.0)	1 (1.4)	
Concurrent medications			
Topical corticosteroids	11 (39.3)	22 (31.9)	0.486
Topical calcineurin inhibitors	8 (28.6)	18 (26.1)	0.802
Oral corticosteroids	5 (17.9)	21 (30.4)	0.205

DAOSD, dupilumab-associated ocular surface disease; EASI, eczema area and severity index; IgE, immunoglobulin E; SD, standard deviation.

^a^ 2 missing data for EASI.

^b^ 35 missing data for IgE.

^c^ 15 missing data for eosinophils.

**FIGURE 1:**
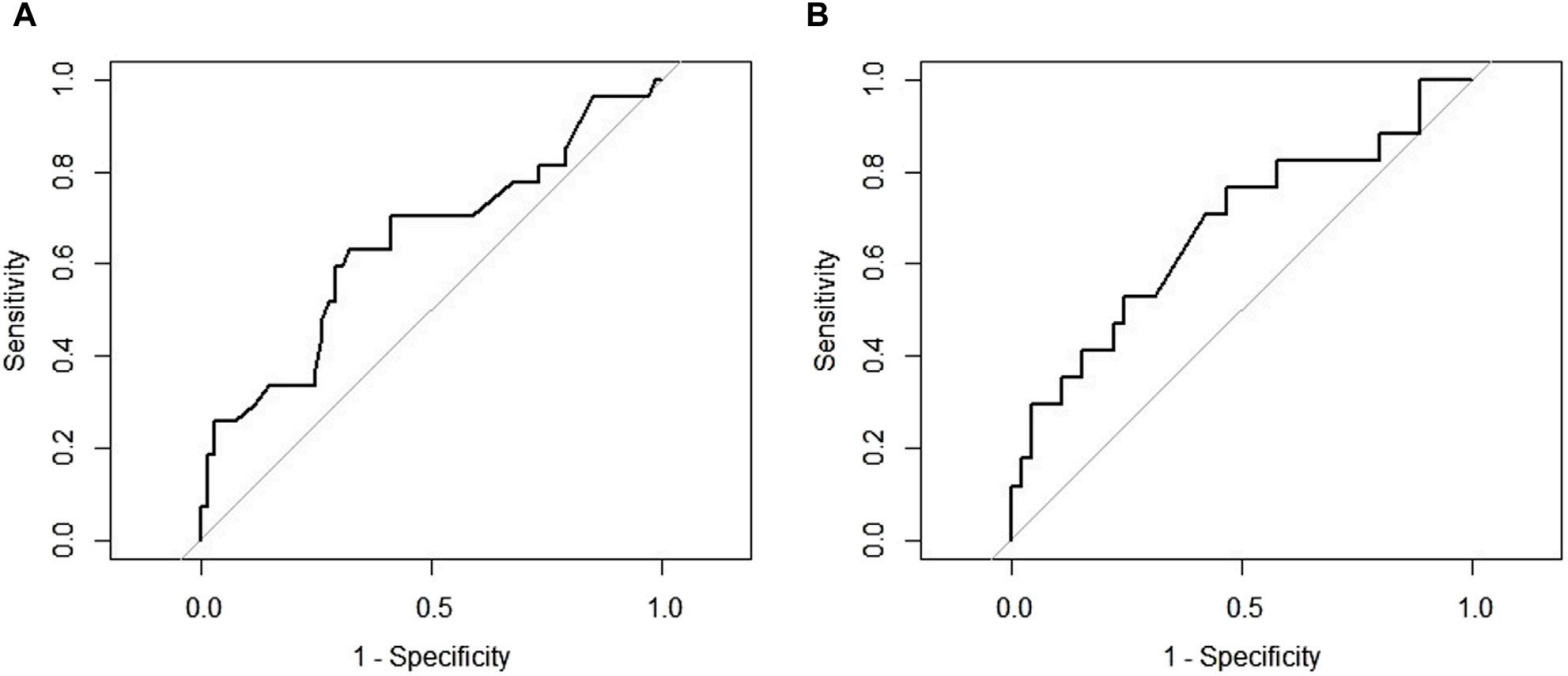
The receiver operating characteristic curve for cut-off points **(A)** Eczema Area and Severity Index at baseline **(B)** Immunoglobulin E at baseline.

Among demographic characteristics, age was significantly associated with DAOSD (*p* = 0.046). Patients who had a history of conjunctivitis showed a higher incidence of DAOSD than individuals without (*p* < 0.001). Compared to those without DAOSD, patients with DAOSD had significantly higher EASI scores (30.3 vs. 26.9, *p* = 0.044) and marginally significantly higher IgE (8,358.1 vs. 3,585.5 (IU/mL), *p* = 0.059) and eosinophils (9.9 vs. 5.8 (%), *p* = 0.053) at baseline. According to ROC curve analysis, a baseline EASI score of 28 satisfied the maximum sensitivity and specificity (sensitivity = 63.0%, specificity = 67.7%; Figure 1A), whereas an IgE of 2,600 (IU/mL) did (sensitivity = 76.5%, specificity = 53.3%; Figure 1B). The occurrence of DAOSD was higher in patients with EASI score ≥28 and IgE ≥2600 IU/mL than the others. No significant difference was observed in previous medications and concurrent medications.

Table 2 presents the associations between clinical factors and DAOSD. A multivariable analysis was performed by incorporating factors significant in univariate analysis, along with age and gender. Due to the large amount of missing data, IgE levels were excluded from the multivariable model. After adjusting the covariates, patients aged 25–39 years and patients aged ≥40 years exhibited a 5.2-fold (95% CI: 1.2–21.7) and 7.4-fold (95% CI: 1.6–35.2) elevated risk of DAOSD compared to patients aged <25 years. Individuals with a previous history of conjunctivitis experienced a 6.6-fold (95% CI: 1.8–23.5) elevated DAOSD risk compared to those without a history. In addition, an EASI score ≥28 at baseline increased DAOSD risk by 3.5 times (95% CI: 1.2–10.2) compared to patients with an EASI score <28. The Hosmer–Lemeshow test results revealed that the models’ fitness was satisfactory (χ^2^ = 6.542, degree of freedom 5, *p* = 0.257). The AUROC of the multivariable analysis was 0.770 (95% CI: 0.653–0.887).

**TABLE 2:** Univariate and multivariable regression analyses to identify predictors for ocular surface disease.

Predictors		Unadjusted OR (95% CI)	Adjusted OR (95% CI)	Score
Women		1.19 (0.47–2.99)		
Age	<25 years	1	1	0
	25–39 years	3.38 (0.99–11.55)	5.16 (1.23–21.75)*	1
	≥40 years	4.82 (1.28–18.18)	7.40 (1.56–35.22)*	2
History of conjunctivitis		7.88 (2.56–24.21)	6.56 (1.83–23.51)**	2
Baseline EASI score ≥28		3.49 (1.38–8.83)	3.47 (1.18–10.20)*	1

CI, confidence interval; EASI, eczema area and severity index; OR, odds ratio. *p < 0.05, **p < 0.01.

In a sensitivity analysis, the missing values for the EASI score, IgE, and eosinophil counts were imputed. After imputation, the distributions of EASI, IgE, and eosinophil in patients with and without DAOSD were consistent with those observed in the main analysis (Supplementary Table S1). When IgE was included in multivariable analysis, it lost statistical significance (*p* = 0.079), with aORs of the other factors remaining similar (Supplementary Table S2).

**FIGURE 2:**
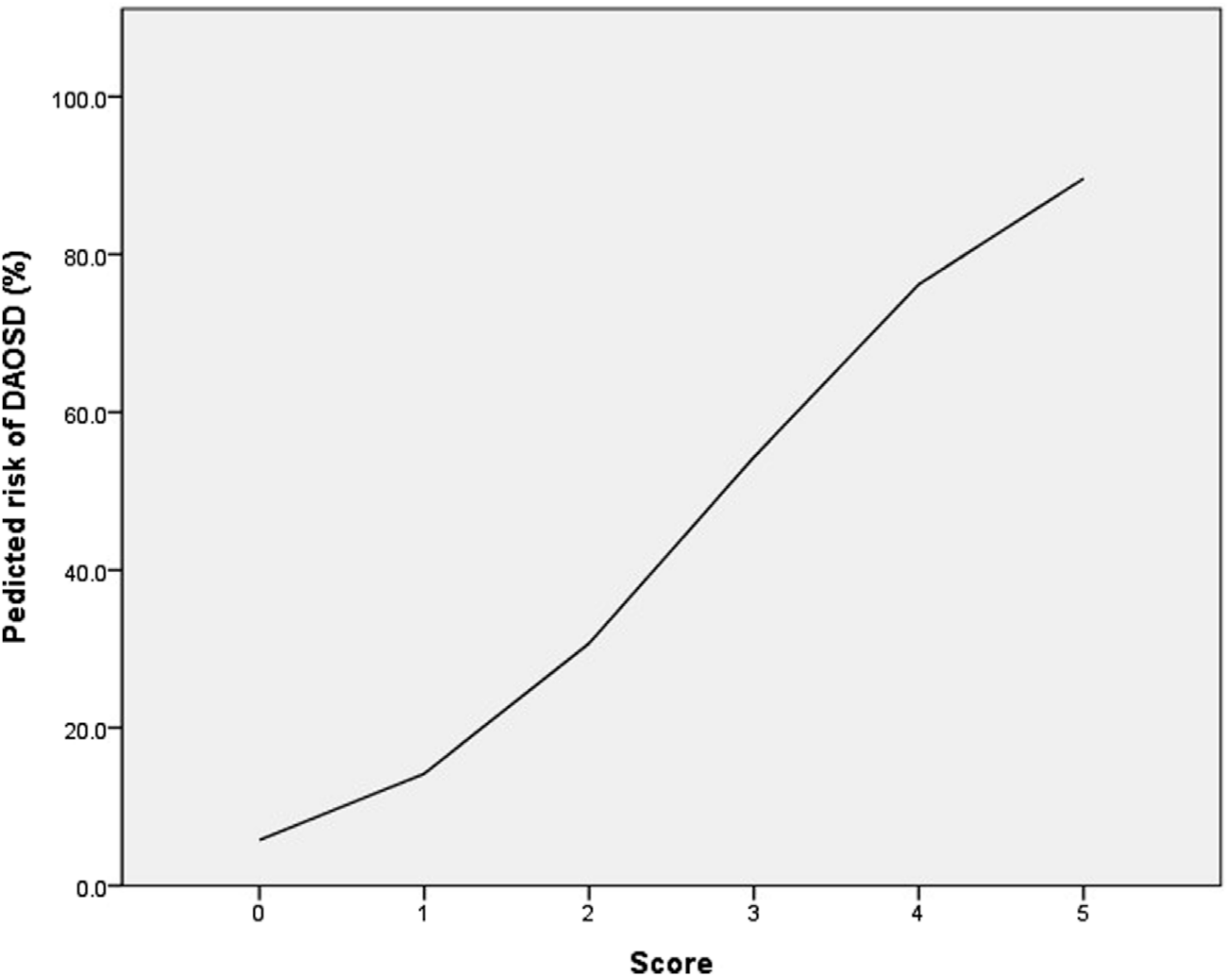
The logistic regression curve of the predicted probability of dupilumab-associated ocular surface disease (DAOSD).

The risk-scoring system was constructed using the significant factors in multivariable analysis. Each variable’s aOR was divided by the aOR of the EASI score ≥28, which served as the reference with the lowest value, and then rounded to the nearest whole number. As a result, the following points were assigned: age <25 years (0 point), age 25–39 years (1 point), age ≥40 years (2 points), history of conjunctivitis (2 points), and baseline EASI score ≥28 (1 point). The risk score of DAOSD in AD patients ranged from 0 to 5 points, and Figure 2 illustrates the risk estimates obtained from the logistic regression curve. The predicted risk of DAOSD for patients with 0, 1, 2, 3, 4, and 5 points was 5.8%, 14.2%, 30.7%, 54.3%, 76.2%, and 89.6%, respectively.

## Discussion

Ocular surface diseases include bacterial/non-bacterial/cicatrizing conjunctivitis, blepharitis, dry eye disease, allergic eye disease, and meibomian gland dysfunction (Achten et al., 2024). Although the pathogenesis of DAOSD is unclear, several hypotheses have been proposed. One pre-existing pathology is associated with inhibiting IL-4 and IL-13 signaling by dupilumab, leading to impaired function of conjunctival goblet cells, ultimately contributing to the development of DAOSD (Achten et al., 2024). The incidence and the onset time of DAOSD have been reported in previous clinical trials and real-world research. Conjunctivitis, one of the ocular surface diseases, has been reported at a higher rate in the dupilumab-treated group in clinical trials (8.6%–22.1%) than in the placebo group (2.1%–11.1%) (van Buuren and Groothuis-Oudshoorn, 2011). In a meta-analysis with real-world data on 908 dupilumab-treated AD patients, the incidence of DAOSD varied from 27.1% to 62.0% (Halling et al., 2021). The most commonly reported adverse drug event was conjunctivitis, accounting for 26.1% (95% CI: 17.8–35.4) (Halling et al., 2021). In addition, DAOSD typically emerged approximately 16 weeks after the initiation of dupilumab therapy (Foley et al., 2022), exhibiting higher incidence rates at week 16 (22.1%) compared to week 52 (17.9%) (Akinlade et al., 2019). These findings were consistent with this study, showing that ocular surface diseases occurred in 28 (28.9%) AD patients receiving dupilumab at an average of 19.7 weeks from the initiation.

According to the findings of this study, patients aged 25–39 years showed increased DAOSD risk by 5.2-fold, and those aged ≥40 years increased by 7.4-fold compared to patients under 25 years. In a survey of 7,044 adult Danes with AD, the prevalence of ocular surface diseases increased with age (Ronnstad et al., 2022). After adjusting for risk factors, lifetime conjunctivitis increased 1.37-fold (95% CI: 1.14–1.66) in adults aged 31–50 years and 1.78-fold (95% CI: 1.42–2.23) in adults aged 51–60 years compared to those aged 18–30 years (Ronnstad et al., 2022). Additionally, a real-world study conducted in the Dubai population showed that adults experienced DAOSD significantly more than patients under 18 years old (80% vs. 20%, *p* = 0.08) (Parmar et al., 2022). These findings suggest that older age should be considered a risk factor.

A history of conjunctivitis has consistently emerged as the most commonly cited risk factor for DAOSD (van Buuren and Groothuis-Oudshoorn, 2011; Nahum et al., 2020; Nettis et al., 2020; Achten et al., 2022), with this study indicating a 6.6-fold increase in associated DAOSD risk. Achten et al. (2022) conducted a prospective multicenter study, revealing an association between DAOSD with a prior history of ocular disease (excluding self-reported episodes) with concomitant ophthalmic medications at enrollment (OR: 5.16, 95% CI: 2.30–11.56). Furthermore, previous studies have shown that AD patients with DAOSD were more likely to have a history of ocular disorders, suggesting that patients who developed DAOSD may have had an undiagnosed ocular disease that was exacerbated by dupilumab (Katsuta et al., 2021; Popiela et al., 2021). Overall, patients with a history of ocular disease would require routine ophthalmologic exams after starting dupilumab (Agnihotri et al., 2019).

The baseline EASI score, the score for AD severity, was another significant risk factor for DAOSD in this study. Conjunctivitis occurred more frequently in patients with severe AD than those with mild to moderate AD in clinical trials (Simpson et al., 2017; Akinlade et al., 2019). Among clinical trials, the CAFÉ trial reported the highest incidence of conjunctivitis at 22.1%, correlating with the highest severity of AD (Akinlade et al., 2019). In a case series of 12 patients who developed conjunctivitis from 142 dupilumab-treated AD patients, all nine patients with severe conjunctivitis had a severe AD assessed by Investigator’s Global Assessment score of 4 before dupilumab treatment (Treister et al., 2018). A prospective multicenter study conducted by Ariens et al. (2021) demonstrated that patients who developed conjunctivitis had higher baseline EASI scores compared to those without [23.4 (IQR 14.4–31.9) vs. 17.7 (IQR 11.5–26.9), *p* = 0.004]. As study patients were limited to severe AD, with the EASI score ranging from 23 to 64, a cut-off of 28 as a threshold based on the ROC curve analysis was suggested. Patients with a baseline EASI score over 28 showed a 3.5-fold increase in DAOSD risk compared to patients with an EASI score below 28. Therefore, close monitoring would be necessary for patients who have high EASI scores when they start dupilumab therapy.

Previous research observed a correlation between elevated levels of type 2 inflammatory biomarkers and a higher incidence of conjunctivitis in patients receiving dupilumab treatment (Akinlade et al., 2019). In addition, serum IgE levels were significantly elevated in AD patients with ocular complications compared to those without (Uchio et al., 1998; Uchida et al., 2020). Similarly, a prospective real-life study of 46 French patients with AD revealed that DAOSD was associated with IgE serum level >1,000 IU/mL at the initiation of dupilumab treatment (OR: 10.6, 95% CI: 1.2–91.3) (Touhouche et al., 2021). This study results were consistent with these findings, showing a significant association between higher IgE levels and an increased risk of DAOSD, thus suggesting a cut-off value of 2,600 IU/mL. However, further studies are needed because an IgE level was not included in the final model due to its missingness.

This study has a few limitations. Firstly, its retrospective nature resulted in the inclusion of a limited number of factors. Further assessment is necessary, including a broader range of biomarkers (e.g., thymus and activation-regulated chemokine, periostin) and patient characteristics (e.g., family history and age at AD onset) as potential factors associated with DAOSD. Second, this study had a relatively small sample size and involved only Koreans, which limits the generalizability of the findings. Prospective multicenter cohort studies and external validation are necessary to validate the findings. Third, DAOSD severity was not assessed, which could be helpful for treatment decisions. Lastly, an elaborate mechanism was not investigated. Nevertheless, to the best of knowledge, this is the first study to construct a risk-scoring system to predict DAOSD.

## Conclusion

This study investigated risk factors for DAOSD and constructed a risk-scoring system using significantly associated variables. These findings can help predict the risk of DAOSD before dupilumab treatment, thereby providing safe treatment for patients with severe AD. Further large cohort studies are needed to validate the findings.

## Data Availability

The raw data supporting the conclusions of this article will be made available by the authors, without undue reservation.
