# Supplementary material for: A risk-scoring system to predict dupilumab-associated ocular surface disease in patients with atopic dermatitis
Source: Front Pharmacol. 2024 Aug 1;15:1425550. doi: 10.3389/fphar.2024.1425550 (PMC11324479; doi:10.3389/fphar.2024.1425550)
Supplement: Supplementary file 1 [file Table1.DOCX]

**Supplementary Table 1.** Biomarkers of patients included after imputation.

| **Variables** | **DAOSD**  **(n = 28)** | **No DAOSD**  **(n = 69)** | ***p*-value** |
| --- | --- | --- | --- |
| EASI score |  |  |  |
| < 28 | 11 (39.3) | 49 (71.0) | 0.004 |
| ≥ 28 | 17 (60.7) | 20 (29.0) |  |
| IgE (IU/ml) |  |  |  |
| < 2600 | 8 (28.6) | 37 (53.6) | 0.025 |
| ≥ 2600 | 20 (71.4) | 32 (46.4) |  |
| Eosinophils (%) |  |  |  |
| < 5.0 | 11 (39.3) | 37 (53.6) | 0.201 |
| ≥ 5.0 | 17 (60.7) | 32 (46.4) |  |

**Supplementary Table 2.** Univariate and multivariable regression analyses to identify predictors for ocular surface disease after imputation.

| Predictors | | Unadjusted OR (95% CI) | Adjusted OR (95% CI) |
| --- | --- | --- | --- |
| Women | | 1.19 (0.47–2.99) |  |
| Age | < 25 years | 1 | 1 |
|  | 25–39 years | 3.38 (0.99–11.55) | 5.07 (1.14–22.45) * |
|  | ≥ 40 years | 4.82 (1.28–18.18) | 8.42 (1.68–42.17) * |
| History of conjunctivitis | | 7.88 (2.56–24.21) | 6.57 (1.82–23.68) ** |
| Baseline EASI score ≥ 28 | | 3.79 (1.51–9.50) | 3.23 (1.07–9.72) * |
| IgE level ≥ 2600 IU/mL | | 2.89 (1.12–7.45) | 2.70 (0.89–8.15) |

CI, confidence interval; EASI, eczema area and severity index; **I**mmunoglobulin E, IgE; OR, odds ratio. **p* < 0.05, ***p* < 0.01
